# X-ROS Signaling Depends on Length-Dependent Calcium Buffering by Troponin

**DOI:** 10.3390/cells10051189

**Published:** 2021-05-13

**Authors:** Sarita Limbu, Benjamin L. Prosser, William J. Lederer, Christopher W. Ward, Mohsin S. Jafri

**Affiliations:** 1 School of Systems Biology and The Krasnow Institute for Advanced Study, George Mason University, Fairfax, VA 22030, USA; limbusarita@gmail.com; 2 Department of Physiology, Pennsylvania Muscle Institute, University of Pennsylvania Perelman School of Medicine, Philadelphia, PA 19104, USA; bpros@mail.med.upenn.edu; 3 Center for Biomedical Engineering and Technology, University of Maryland School of Medicine, Baltimore, MD 20201, USA; jlederer@som.umaryland.edu; 4 Center for Biomedical Engineering and Technology and Department of Orthopaedics, University of Maryland School of Medicine, Baltimore, MD 20201, USA; ward@umaryland.edu

**Keywords:** heart, reactive oxygen species, calcium, blebbistatin, computational model

## Abstract

The stretching of a cardiomyocyte leads to the increased production of reactive oxygen species that increases ryanodine receptor open probability through a process termed X-ROS signaling. The stretching of the myocyte also increases the calcium affinity of myofilament Troponin C, which increases its calcium buffering capacity. Here, an integrative experimental and modeling study is pursued to explain the interplay of length-dependent changes in calcium buffering by troponin and stretch-activated X-ROS calcium signaling. Using this combination, we show that the troponin C-dependent increase in myoplasmic calcium buffering during myocyte stretching largely offsets the X-ROS-dependent increase in calcium release from the sarcoplasmic reticulum. The combination of modeling and experiment are further informed by the elimination of length-dependent changes to troponin C calcium binding in the presence of blebbistatin. Here, the model suggests that it is the X-ROS signaling-dependent Ca^2+^ release increase that serves to maintain free myoplasmic calcium concentrations during a change in myocyte length. Together, our experimental and modeling approaches have further defined the relative contributions of X-ROS signaling and the length-dependent calcium buffering by troponin in shaping the myoplasmic calcium transient.

## 1. Introduction

The mechanical stretch of cardiac myocytes activates NADPH oxidase 2 (NOX2) which generates reactive oxygen species (ROS) signals that target ryanodine receptor type 2 (RyR2) to activate Ca^2+^ spark rate [1,2]. While this mechano-chemo transduction pathway, known as X-ROS signaling, is providing a new understanding of cardiomyocyte function in health and disease [1,2,3,4,5,6], the molecular details of this pathway remain to be fully defined.

We have developed a computational model to dissect the mechanism of X-ROS signaling and predict the impact it has on normal and pathological physiology [5]. One prediction of the model was that that stretching just prior to the action potential, such as that which might occur by the filling of the ventricles, optimally potentiates action potential-induced Ca^2+^ release. In fact, in cardiac myocytes, stretching has been shown to increase the magnitude of the Ca^2+^ and force transients during pacing [7,8].

Troponin C (TnC) is the Ca^2+^ binding subunit of the troponin complex that is also comprised of an inhibitory subunit, troponin I (TnI) and a tropomyosin binding subunit, troponin T (TnT). TnC has two COOH-terminal high-affinity Ca^2+^ binding sites (site III and IV) that can also bind Mg^2+^ (also known as Ca^2+^-Mg^2+^ sites) and two NH_2_-terminal low-affinity Ca^2+^ binding sites (site I and II) that are Ca^2+^-specific. Only the Ca^2+^-specific sites in TnC can trigger muscle contraction [9,10] and in the case of cardiac TnC, only site II can initiate muscle contraction [11,12,13].

A reduction in force or sarcomere length reduces the Ca^2+^ sensitivity of cardiac troponin C (cTnC) [14]. The changes in Ca^2+^ sensitivity have been attributed to a change in the number of actin–myosin cross-bridges, which may arise from length-dependent changes in the spacing between actin and myosin filaments as well as structural rearrangements in the thin and thick filaments [15,16]. An increase in sarcomere length decreases the separation between actin and myosin and causes a change in troponin structure.

In this manuscript, we pursue an integrative experimental and computational modeling approach to improve our understanding of X-ROS signaling and its effect on excitation–contraction coupling by considering length-dependent changes in troponin Ca^2+^ sensitivity. During systole, the sarcoplasmic reticulum (SR) releases Ca^2+^ ions that bind to troponin C (TnC) present on actin (the thin filament). The binding of Ca^2+^ ions to TnC changes the conformation of TnC, exposes myosin binding sites on actin and thus initiates myocyte contraction. Crossbridge formation between actin and myosin increases the Ca^2+^ binding affinity of TnC and contributes to increasing the force of contraction of the cardiac myocytes.

The combination of experiments and modeling suggest that stretching offers two antagonistic effects on the level of measured [Ca^2+^]_i_ during excitation–contraction coupling. Stretching increases the affinity of troponin for Ca^2+^, increasing Ca^2+^ buffering and force generation, i.e., buffering by troponin promotes the contraction. On the other hand, X-ROS signaling provides an increase in Ca^2+^ mobilization during stretching that contributes to increases in the measured [Ca^2+^]_i_ level and hence may contribute to contraction. It does this by offsetting a possible reduction in measured [Ca^2+^]_i_ due to the buffering of Ca^2+^ by troponin.

## 2. Materials and Methods

### 2.1. The Model

**Figure 1:**
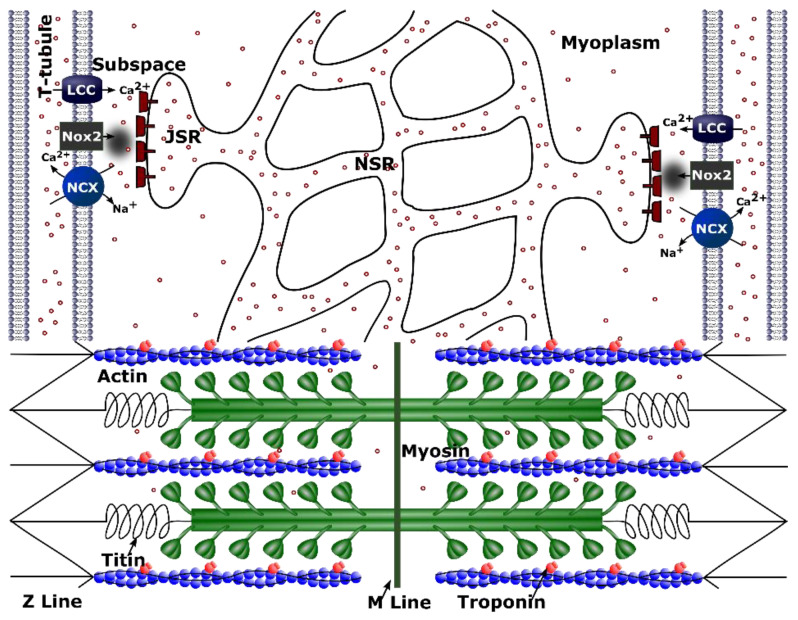
Schematic figure for calcium handling model. ROS are produced by NOX2 close to the Ca^2+^ release site as a result of stretching. Only one of the 20,000 Ca^2+^ release units from the model of a single cell is shown. Purple circles indicate calcium ions (Ca^2+^). Other abbreviations: NCX—Na^+^-Ca^2+^ exchanger; PMCA—plasmalemmal Ca^2+^-ATPase; I_Na_—sodium current; I_to_—transient outward potassium current; I_K1_—potassium current; LCC—L-type Ca^2+^ current; RYR2—ryanodine receptor channel; ROS—reactive oxygen species; NOX2—NADPH oxidase type 2; CSQ—calsequestrin; JSR—junctional sarcoplasmic reticulum; NSR—network sarcoplasmic reticulum; CaM—calmodulin.

We previously developed a model for excitation–contraction coupling in the rat ventricular myocyte that included a novel 4-state formulation for the RyR2 that simulates the increase in open probability of an RyR2 channel due to its oxidation by stretch-induced ROS (Figure 1) [5]. To improve upon this model, we now include the following: (1) the addition of the low-affinity Ca^2+^ binding of sites on troponin and (2) stretch-dependent changes in Ca^2+^ affinity for both the low- and high-affinity Ca^2+^ binding sites on troponin.

Our previous work included only the high-affinity binding sites of the troponin buffer which are considered to be occupied by Mg^2+^ or Ca^2+^ most of the time. Therefore, a more realistic model for binding of Ca^2+^ to troponin buffer, described by Jafri et al. [17], that considers both the high-affinity and low-affinity binding sites is used. The equations describing troponin dynamics are:

(1) $$J_{trpn-high}=k_{trpn-high}^{+}{\left[Ca^{2+}\right]}_{i}\left({\left[trpnT\right]}_{high}-{\left[trpnCa\right]}_{high}\right)-k_{trpn-high}^{-}{\left[trpnCa\right]}_{high}$$

(2) $$J_{trpn-low}=k_{trpn-low}^{+}{\left[Ca^{2+}\right]}_{i}\left({\left[trpnT\right]}_{low}-{\left[trpnCa\right]}_{low}\right)-k_{trpn-low}^{-}{\left[trpnCa\right]}_{low}$$

where $k_{trpn-high}^{+}$ and $k_{trpn-low}^{+}$ are the rates of Ca^2+^ on, and $k_{trpn-high}^{-}$ and $k_{trpn-low}^{-}$ are the rates of Ca^2+^ off for high-affinity and low-affinity binding sites of troponin, respectively. ${\left[trpnT\right]}_{high}$ and ${\left[trpnT\right]}_{low\;}$ are the total myoplasmic content of high-affinity and low-affinity binding sites of troponin, respectively, and ${\left[trpnCa\right]}_{high}$ and ${\left[trpnCa\right]}_{low}$ are the concentrations of Ca^2+^ bound to high-affinity and low-affinity troponin binding sites, respectively. Added to this model is the increase in Ca^2+^ sensitivity of cTnC due to an increase in the sarcomere length. To represent this, we increased the opening rates ($k_{trpn}^{+}$) for both the high-affinity as well as low-affinity binding sites by 20% to simulate when the myocyte is stretched 10%. The $k_{trpn}^{+}$ rate returns to its initial value once the myocyte is released from the stretch. The increase in the opening rates is constrained by fitting nonlinear curves to the experimental data that have a lower root mean squared error than linear fits [18].

When stretched, the actual amount of ROS produced ($J_{ROSProduction}^{k}$) by NOX2 in each subspace is given by the following set of four time-dependent equations

$$(i)\;J_{ROSProduction}^{k}=ROS_{preStr}+\frac{\left(0.0002-ROS_{preStr}\right)}{0.12}\;t\;\mathrm{for}\;t\;<\;t_{Str}$$

$$(\mathrm{ii})\;J_{ROSProduction}^{k}=0.000036\;(t^{2})-0.000122\;(t)+0.000214\;t_{Str}\;\leq \;t\;<\;1.5\;s$$

$$(\mathrm{iii})\;J_{ROSProduction}^{k}=0.000001\;(t^{2})-0.0000152\;(t)+0.0001326716\;1.5\;s\;\leq \;t\;<\;4\;s$$

$$(\mathrm{iv})\;J_{ROSProduction}^{k}=0.0001259488\;(exp^{-0.09t})\;t\;\geq \;4\;s$$

where $ROS_{preStr}$ is the rate of stretch-induced ROS production just before the stretching is carried out (=$J_{ROSProduction}^{k}$ at the time of stretching) and is equal to 0 s^−1^ in the resting condition, and $t_{Str}$ is the time taken to reach the peak stretching. The coefficients and order of the equations were selected such that the outcome would resemble the trend of the ROS production rate obtained experimentally by Prosser et al. [1]. The case when myocyte length is returned to normal is referred to here as “release of stretch”. If *time_after_release* ≤ $time_{ROSEnd}$ (time taken for stretch-induced ROS production to cease after the release of stretch) then

$$(v)\;J_{ROSProduction}^{k}=ROS_{prerelease}\left(1-\frac{time\_after\_release}{time_{ROSEnd}}\right)$$

where $ROS_{prerelease}$ is the rate of ROS production just before the release of stretch is carried out (=$J_{ROSProduction}^{k}$ at the time of release of stretch).

### 2.2. Experimental Methods

#### 2.2.1. Rodent Models

Animal care and procedures were approved and performed in accordance with the standards set forth by the University of Maryland, Baltimore Institutional Animal Care and Use Committee and the Guide for the Care and Use of Laboratory Animals published by the US National Institutes of Health (NIH Publication, 8th Edition, 2011).

#### 2.2.2. Materials

Gp91ds-tat was purchased from Anaspec (Fremont, CA, USA) and used at 1 µM. Blebbistatin was purchased from Abcam and used at 3 µM.

#### 2.2.3. Cardiomyocyte Isolation

Adult, male, Sprague–Dawley rats (8–16 weeks) were terminally anaesthetized by the injection of pentobarbital (200 mg/kg), followed by the excision of the heart and enzymatic isolation of ventricular myocytes as previously described (Mitra and Morad Am J Physiol 1985). Cardiomyocytes were stored in a normal Tyrode’s solution containing (in mmol/L): NaCl 140, KCl 5, CaCl_2_ 1.8, MgCl_2_ 0.5, HEPES 5, Glucose 5, NaH_2_PO_4_ 0.33. Experiments were performed at room temperature, 22 °C.

#### 2.2.4. Cardiomyocyte Stretch Experiments

Experiments were performed in custom fabricated cell chambers (Four-hour Day Foundation, Towson, MD, USA) mounted on a Zeiss LSM 510 inverted confocal microscope with a 40 Oil 1.2 NA objective. The [Ca^2+^]_i_ transient was evaluated with Fluo-4 by 15 min incubation with 2 mM Fluo-4-AM ester and 0.01% Pluronic F127. Cells were allowed an additional 10 min for de-esterification and then scanned using a 488 nm argon ion laser in confocal line-scan mode at 1.92 ms per line. Cells were paced to steady state using 0.5 Hz field stimulation through platinum electrodes. After 20 s of 1 Hz stimulation, all cells showed steady state transients and contractions. At this point, five calcium transients before, during and after the release of stretching were acquired. This protocol was repeated twice in each myocyte, and data were pooled for analysis.

The cardiomyocyte stretch was performed as previously described in Prosser et al., 2011. Briefly, glass microrods were coated with a biological adhesive, MyoTak^®^ (IonOptix, Milton, MA, USA). One glass microrod was connected to a force transducer (403A-CF, Aurora Scientific, Aurora, ON, Canada), and the other to a length controller (World Precision Instruments, Sarasota, FL, USA). Myocytes were attached at both ends by gently pressing down with the MyoTak-coated microrod and then lifting the cell from the chamber bottom. Axial stretching was applied by movement of the length controller in response to variable voltage output. Myocytes were subjected to two stretch-release trials, with a 30–60 s rest allowed between trials.

## 3. Results

**Figure 2:**
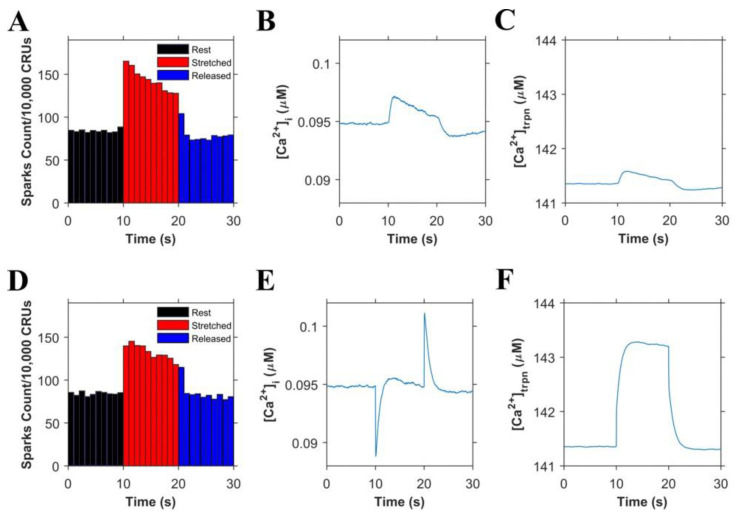
Simulation results (n = 20 simulations) in resting cells. (**A**–**C**). Simulations in which troponin’s affinity to Ca^2+^ does not increase during the stretching of cardiomyocytes: **A**. Ca^2+^ sparks histograms for 1 s bins. **B**. Concentration of Ca^2+^ in the myoplasm ([Ca^2+^]_i_). **C**. Concentration of Ca^2+^ bound to troponin ([Ca^2+^]_trpn_). (**D**–**F**). Simulations in which troponin’s affinity to Ca^2+^ increases during the stretching of cardiomyocytes: **D**. Ca^2+^ sparks histograms for 1 s bins. **E**. Concentration of Ca^2+^ in the myoplasm ([Ca^2+^]_i_). **F**. Concentration of Ca^2+^ bound to troponin ([Ca^2+^]_trpn_).

Informed by our previously published results, we updated our model and performed numerical experiments using model simulations to isolate and dissect the mechanisms of stretching-dependent Troponin C Ca^2+^ affinity on EC coupling. Similar to our previous experimental findings [1], this improved model of X-ROS signaling produces a ~two-fold increase in Ca^2+^ spark frequency upon myocyte stretch (Figure 2A) that resolves to baseline when returned to resting length. Additionally, the model simulation shows a very small, transient rise in the myoplasmic Ca^2+^ concentration ([Ca^2+^]_i_) (Figure 2B) as we previously showed experientially [5]. Model simulations revealed the contribution of Troponin C buffering (Figure 2C) occurred independent of length-dependent changes in the affinity of troponin for Ca^2+^.

The next set of numerical experiments interrogated the previous report of a 20% increase in troponin C Ca^2+^ affinity driven by a 10% increase in length [18]. Here, we observed that with an acute stretching, Ca^2+^ spark frequency increases abruptly by ~1.7-fold which closely recapitulates our experimental data [1] (Figure 2D). While stretching also elicits an abrupt increase in troponin Ca^2+^ sensitivity which predisposes a sudden decrease in [Ca^2+^]_i_, the simultaneous increase in X-ROS elicits Ca^2+^ sparks, which offsets the effect (Figure 2E). Furthermore, when the myocyte length is returned to resting length, the abrupt decrease in the troponin sensitivity drives a transient elevation of [Ca^2+^]_i_, which is offset by the immediate decrease in Ca^2+^ spark frequency. Consistent with this behavior is the numerical result of a sustained stretch, which would maintain the increased affinity of Troponin for Ca^2+^ [Ca^2+^]_trpn_ throughout the stretched period (Figure 2F).

Cardiomyocyte stretching therefore activates at least two mechanisms: (1) X-ROS signaling that increases SR Ca^2+^ release (via spark activation, Figure 2A) resulting in increased [Ca^2+^]_i_ (Figure 2B), and (2) an increase in the Ca^2+^ binding affinity of troponin which increases Ca^2+^ buffering. To extend these concepts to the global Ca^2+^ transient, experiments were performed in electrically stimulated myocytes.

**Figure 3:**
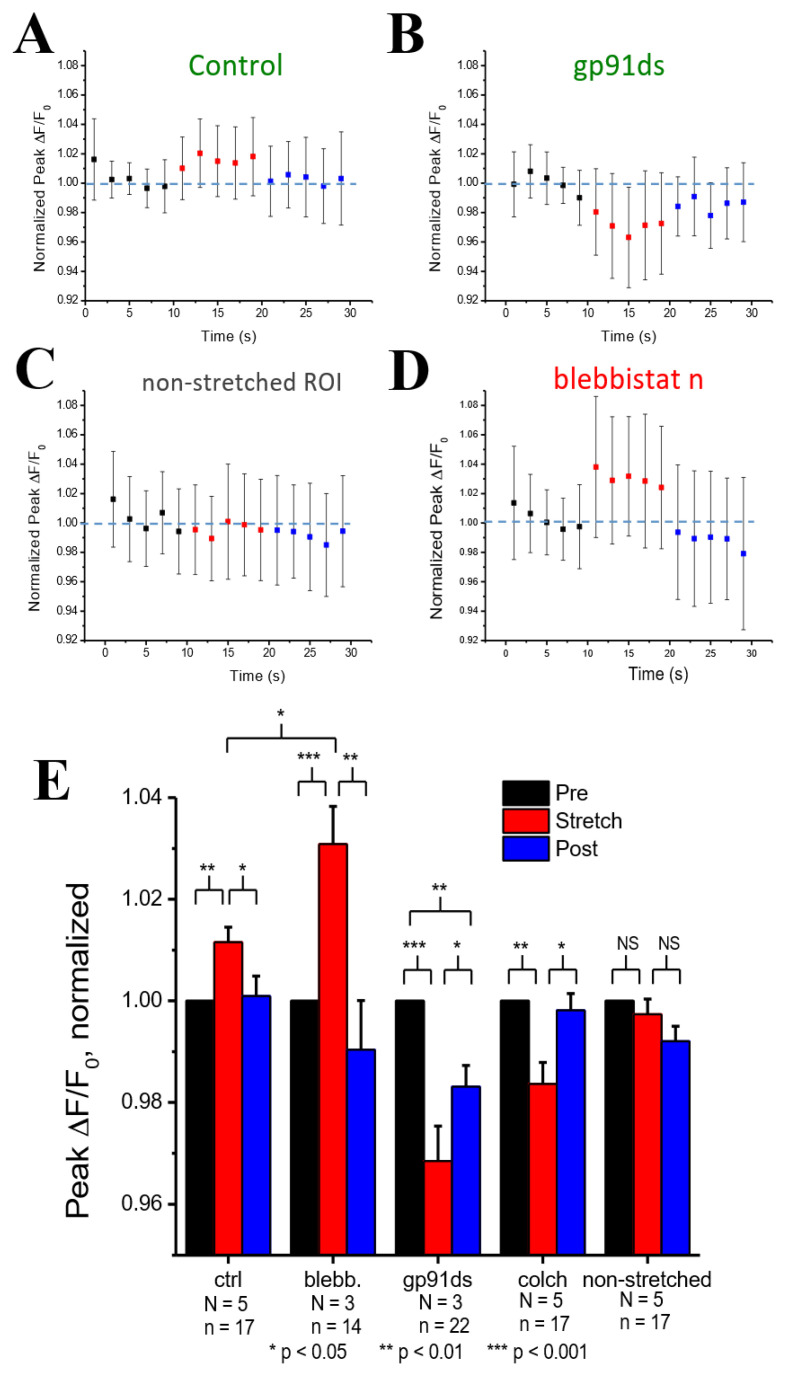
Experimental results for the normalized peak ΔF/F0 for the concentration of Ca^2+^ in the myoplasm ([Ca^2+^]_i_) with 0.5 Hz stimulus when the cardiomyocyte is at rest (black), stretched (red) and released after the stretch (blue) **A**. Under control condition. **B**. When blebbistatin is used which inhibits the actin–myosin bridge formation. **C**. When gp91ds is used which inhibits NOX2 from producing stretch-dependent ROS. **D**. In a non-stretched region of control myocytes, and **E**. the calculated average of peak ([Ca^2+^]_i_) under each subgroup (rest, stretched and released) of each group (control, blebbistatin, gp91ds, colchicine and non-stretched). N is the number of animals and n is the number of cells. Statistical comparisons were carried out using the paired *t*-test.

Figure 3A shows experiments in cardiomyocytes field stimulated at 1 Hz in which the peak Ca^2+^ concentration (ΔF/F_0_) was quantified before, during, and after, stretching to one-half of the cardiomyocyte. Transients within the 10 s duration of each period were averaged and normalized to the period before stretching (Figure 3E). Here, we show that stretching elicited a small rise in the peak [Ca^2+^]_i_ of the stretched (Figure 3A) vs. non-stretched (Figure 3D) region that was rapidly reversed upon return to its unstretched level. Given that actin–myosin cross-bridge formation is required for the length-dependent change in TnC Ca^2+^ sensitivity [19], we sought to disrupt cross-bridge formation as an empirical test of Troponin C’s effect.

Blebbistatin is a chemical that inhibits actin and myosin crossbridge formation by lowering the affinity of myosin binding to actin. Consistent with this action, the Ca^2+^ binding affinity of troponin has been reported to decrease with increasing blebbistatin and increase with an increase in sarcomere length [18]. Monitoring [Ca^2+^]_i_ in blebbistatin-treated myocytes, we show that stretch elicits a greater increase in [Ca^2+^]_i_ compared to stretch in untreated controls (Figure 3B,E). To dissect the contribution of X-ROS to the regulation of the global Ca^2+^ transient, we treated myocytes with gp91ds-TAT (a peptide inhibitor of NOX2), or colchicine (to destabilize the microtubule network, see [1]. The X-ROS signaling phenomena is the increased activation of RyR leading to increased Ca^2+^ sparks induced by stretching the myocyte. Stretching the myocytes deforms the microtubular network that activates NOX2 to produce ROS. Previous studies with DCF fluorescence showed that ROS was increased during stretching [6]. The DCF fluorescence measurements were not repeated here. Colchicine was used to reduce the microtubular network and hence the activation of NOX2. X-ROS suppression with either method significantly decreased peak of the global Ca^2+^ transient with stretch, essentially unmasking the increase in Troponin C Ca^2+^ affinity with stretch (Figure 3C,E).

**Figure 4:**
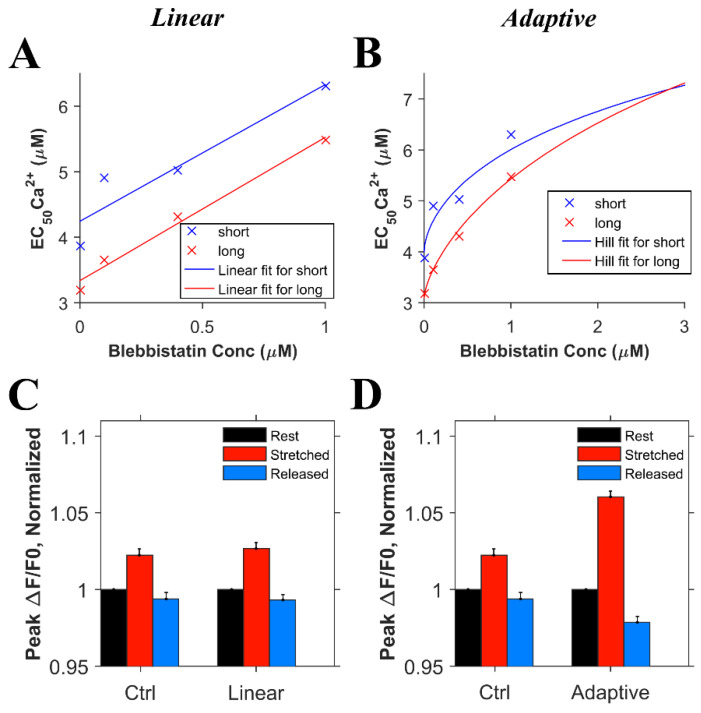
The effects of blebbistatin on the Ca^2+^ binding affinity of troponin. **A**. A linear fit suggests that the length-dependent difference in binding affinity remains relatively constant as a ~20% decrease in EC50. The × symbols represent the experimental data from Farman et al. [18] and the lines the fit. Blue denotes short sarcomere lengths, while red denotes long sarcomere lengths (mean squared error = 0.085) **B**. An adaptive fit using the Hill equations show that the length-dependent difference in the binding affinity decreases and eventually disappears with increasing blebbistatin concentration (mean squared error = 0.062). **C**. Simulation results (n = 20 simulations) for the comparison between control and adaptive cases. **D**. Simulation results (n = 20 simulations) control and adaptive cases.

**Figure 5:**
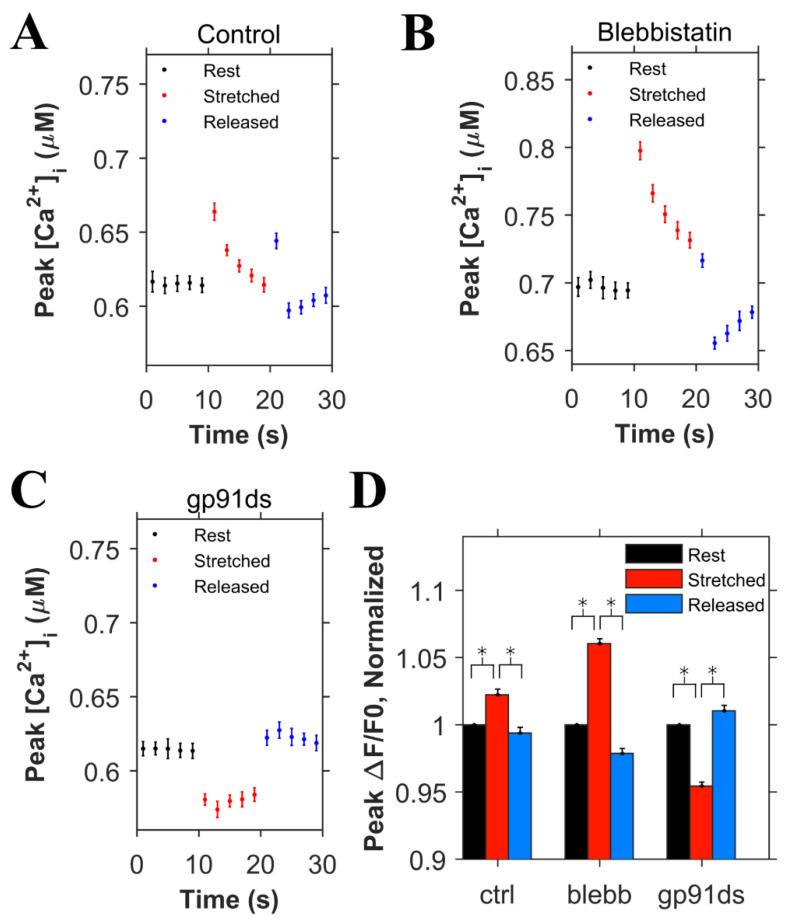
Simulation results (n = 20 simulations) for the peak concentration of Ca^2+^ in the myoplasm ([Ca^2+^]_i_) with 0.5 Hz stimulus when the cardiomyocyte is at rest length (black), stretched (red) and released after the stretch (blue). **A**. Under control condition. **B**. When blebbistatin which inhibits the actin myosin bridge is used. **C**. When gp91ds is used which inhibits NOX2 from producing stretch-dependent ROS. **D**. The peak ([Ca^2+^]_i_) under each subgroup (rest, stretched and released) of each group (control, blebbistatin and gp91ds) is averaged, its corresponding ΔF/F0 is calculated and normalized. * *p* < 10^−14^.

Informed by these new experimental results on the global transient, we conducted numerical experiments. In order to model the effects of blebbistatin, experimental data quantifying the effects of blebbistatin on the Ca^2+^ affinity of troponin were considered. According to the observation by Farman et al. [18], there was a decrease in calcium sensitivity with an increase in the blebbistatin concentration. However, using the approximately linear fit suggested in the paper, the length-dependent differences in the calcium binding affinity of troponin remained relatively constant between 0 and 1 µM blebbistatin, as seen by the difference between the two lines in Figure 4A. On the other hand, fitting the EC_50_ data for calcium sensitivity to the Hill equation (adaptive fit) gave a better least squares error than a linear fit for both the short and long sarcomere lengths, the result being that length-dependent differences disappear as the blebbistatin concentration increases (Figure 4B). To assess which of these interpretations was more likely, we carried out two sets of simulations considering the data for the linear and adaptive cases. For the first set of simulations, we considered that the length-dependent change in the calcium binding affinity of troponin is the same in the presence or absence of blebbistatin (Figure 4C, linear) and hence, when the myocyte is stretched, the $k_{trpn-high}^{+}$ and $k_{trpn-low}^{+}$ increases by 20% in both the presence of blebbistatin and the absence of blebbistatin (Figure 5D, ctrl). For the second set of simulations, we considered that blebbistatin (at 3 µM concentration) would inhibit the length-dependent change in the calcium binding affinity of troponin (Figure 4D, adaptive) and hence, when the myocyte is stretched, the $k_{trpn-high}^{+}$ and $k_{trpn-low}^{+}$ remain unchanged. The results of the second set of simulations (i.e., adaptive) resembled the experimental data (Figure 3E, blebb) more closely than the results of the first set of simulations did (i.e., linear least sq.).

Using the adaptive fit described above, the simulation data were used to simulate the experiments displayed in Figure 3. In the control simulations (Figure 5A), the $k_{trpn}^{+}$ rates for both the high and low affinity binding sites were increased by 20% when the myocyte was stretched to simulate the effects of length-dependent change in Ca^2+^ affinity displayed by troponin [18]. With this affinity increase, there was a small transient increase in [Ca^2+^]_i_ (Figure 5A). Removal of this increase in affinity to simulate the presence of blebbistatin (adaptive case) resulted in the expected almost doubling of the increase [Ca^2+^]_i_ level during the stretching (Figure 5B) over control stretching (Figure 5A), similar to experimental data. Additionally, the block of X-ROS signaling to simulate the presence of gp91ds shows the buffering effect of troponin that increases during stretching (Figure 5C). The peak [Ca^2+^]_i_ during the stimulation every 2 s were averaged for rest, stretched and released conditions, and corresponding ΔF/F0 values were calculated, and normalized (Figure 5D). These data sets show results similar to experiments (Figure 3E). When stretched, the peak normalized ΔF/F0 increased by 2.2% in the control case whereas by 6% when there was no change in the Ca^2+^ binding affinity of troponin (blebb). When no X-ROS was produced (gp91ds), the peak normalized ΔF/F0 decreased by 4.6%. The agreement of the experiment and simulation indicate the relative contributions of X-ROS signaling and myofilament calcium buffering by troponin to stretching-induced changes in [Ca^2+^]_i_ transient amplitudes.

**Figure 6:**
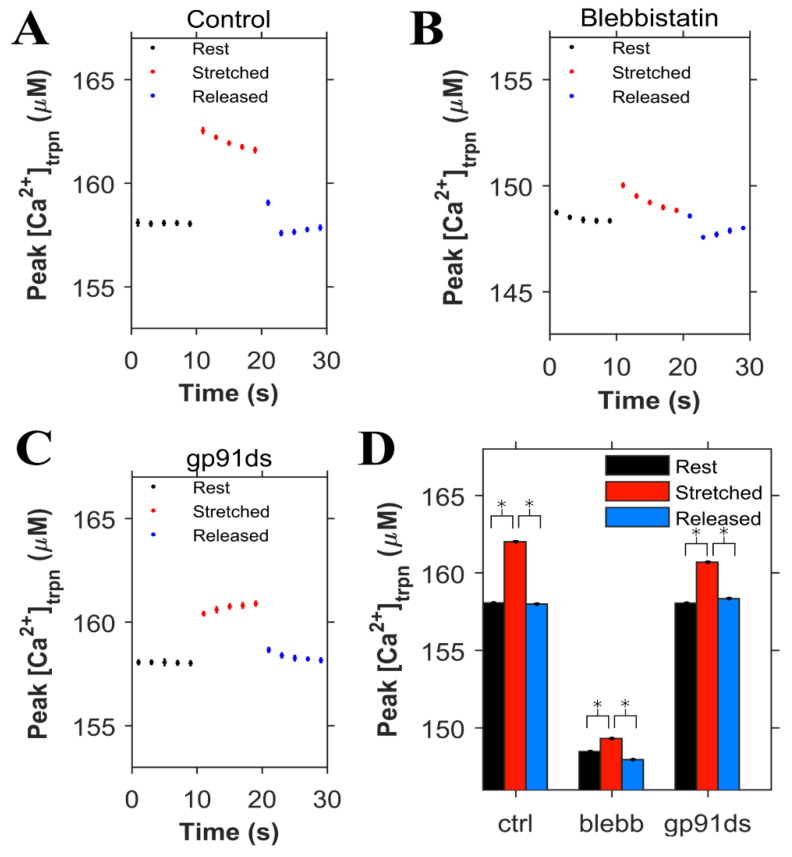
Simulation results (n = 20 simulations) for cardiomyocytes paced at 0.5 Hz (same simulations as Figure 5). The peak value of Ca^2+^ bound to troponin ([Ca^2+^]_trpn_) is shown when the cardiomyocyte is at rest (black), stretched (red) and released after the stretch (blue). **A**. Under control condition. **B**. When blebbistatin is used which inhibits the actin myosin bridge. **C**. When gp91ds is used which inhibits NOX2 from producing stretch-dependent ROS. **D**. The calculated corresponding average of peak ([Ca^2+^]_trpn_) under each subgroup (rest, stretched and released) in each group (control, blebbistatin and gp91ds). * *p* < 10^−22^.

The concentration of Ca^2+^ bound to troponin ([Ca^2+^]_trpn_) was determined in each condition (control, blebb and gp91ds), displayed in Figure 6, using the same set of simulations as in Figure 5. During the 0.5 Hz pacing protocol, the peak [Ca^2+^]_trpn_ was 158 µM in the control condition, increased with myocyte stretch, and returned to its initial level when the cell was returned to resting length (Figure 6A). Numerical experiments with blebbistatin revealed a significant drop in the peak [Ca^2+^]_trpn_ at baseline and with stretch consistent with the reduced troponin Ca^2+^ binding affinity (Figure 6B). In contrast is our modeling of gp91ds, which inhibits stretch-dependent X-ROS independent of the stretch-dependent increase in [Ca^2+^]_trpn_ (Figure 6C).

## 4. Discussion

We conducted an integrated series of simulations and experiments to define the interplay between X-ROS signaling and the length-dependent changes in the Ca^2+^ binding affinity of troponin. The initial model predictions suggested that with stretching, the transient increase in myoplasmic [Ca^2+^] activated by X-ROS signaling would be reduced by the length-dependent increase in buffering by troponin due to the increase in Ca^2+^ binding affinity. The model also suggested that in the contracting myocyte, this behavior would result in a rapid and transient decrease in [Ca^2+^]_i_ due to length-dependent troponin sensitization followed by a small transient rise in [Ca^2+^]_i_ due to X-ROS-dependent activation of the RyR. Upon relaxation, there would then be a small transient increase in myoplasmic [Ca^2+^] due to the reversal of troponin sensitization. In fact, these predictions were consistent with the observation by Backx and ter Keurs, who reported a transient increase in myoplasmic Ca^2+^ at the end of a twitch that they attributed to length-dependent changes in troponin dynamics [20].

Experiments to dissect the relative contribution of X-ROS signaling from myofilament calcium buffering during stretch used blebbistatin to reduce the length-dependent effects on Ca^2+^ binding by troponin. While recent results suggest that blebbistatin may reduce troponin Ca^2+^ affinity independent of having an impact on length-dependent activation [18], this conclusion was inconsistent with the experimental data presented (see Figure 3). Our examination of these results leads us to suspect that the use of a linear fit to describe the data underlies this inconsistency. Support for this possibility comes from our use of an adaptive fit in which the reduction in troponin Ca^2+^ binding affinity with increasing blebbistatin was accompanied by a reduction in and eventual elimination of length-dependent differences in binding affinity. In fact, the mean squared error of a linear vs. adaptive fit revealed the latter to be superior for the experimental data presented in Figure 4. Furthermore, the adaptive fit enabled our model to faithfully reproduce the experimental result. Together, both the experimental results and model simulations suggest that blebbistatin acts in a concentration-dependent manner to progressively reduce, then eventually eliminate, the length dependence of Ca^2+^ binding to troponin.

The new model offers insight into the interplay between X-ROS signaling and myofilament calcium buffering. The interaction between the regulation of stretch-dependent Ca^2+^ spark behavior and cytosolic [Ca^2+^] can be observed. During stretching, the increase in troponin Ca^2+^ affinity—if unopposed—would lead to a transient reduction in myoplasmic [Ca^2+^]. However, if there is a simultaneous rapid burst of X-ROS signaling that increases the sensitivity of RyR2 to activation by Ca^2+^-induced Ca^2+^ release (CICR), a rapid activation of Ca^2+^ spark activity could occur. Given the importance of length-dependent myofilament Ca^2+^ binding and activation in the regulation of cardiac contractility, the burst of X-ROS signaling with stretch appears to be an important mechanism that offsets the consequences of increased Ca^2+^ affinity by troponin. In contrast, during the return of the myofilaments to resting length from stretching, the kinetics of the decrease in Ca^2+^ binding to troponin and the decrease in Ca^2+^ spark activity due to the decline of X-ROS signaling are critical to the time-course of [Ca^2+^]_i_. Despite the impact of the X-ROS burst rapidly waning, the acute reduction in the Ca^2+^ buffering by troponin may transiently increase myoplasmic [Ca^2+^]. If so, CICR would increase and there would be a brief delay in the return of Ca^2+^ spark activity to the levels before stretching (Figure 2D).

The interactions of X-ROS signaling, and myofilament calcium buffering was further interrogated by using blebbistatin to ablate length-dependent troponin sensitization of troponin and gp91ds to inhibit NOX2 and thus X-ROS signaling. Consistent with blebbistatin acting to inhibit the length-dependent increase in affinity of troponin for Ca^2+^, its effect in both experiments and model simulations was to increase stretch-dependent Ca^2+^ spark activation and hasten the return of Ca^2+^ spark activity upon a return to resting length. In the case of inhibiting X-ROS with gp91ds-TAT, both the experiment and model reveal a reduction in [Ca^2+^] with stretching which is consistent with a decrease in X-ROS-dependent RyR2 activation in the face of the increased troponin Ca^2+^ affinity during stretch. In contrast, is the impact of X-ROS inhibition during the return to resting length following stretching. Here, the model returns Ca^2+^ spark activity to the levels before stretching while the experimental system does not. We take this incongruency between experiment and model results as potential evidence of a target or effect of X-ROS signaling not yet included in the model.

Studies have suggested that in cardiac myocytes, there is an abrupt increase in force after stretching (rapid response) and a slower response that evolves over several minutes (slow force response). A potential mechanism for the slow force response has been suggested, including the activation of stretch-activated non-specific cation channels and paracrine/autocrine signaling. The slow force response has been shown to be helpful in normal function and detrimental in diseases such as heart failure [21]. In the rapid response, when the heart muscle is stretched, there is an increase in force seen in the same action potential. Previous studies have suggested that this is due to a decrease in myofilament overlap and an increase in the Ca^2^ sensitivity of the myofilaments (i.e., troponin) [7]. Our previous studies have shown that the X-ROS mechanism can contribute to both the rapid response and the slow frequency response [5]. The X-ROS mechanism causes stretching just prior to a contraction to result in the optimal augmentation of contraction. This response is attenuated upon subsequent beats but does contribute to be helpful in normal function. With oxidative stress, which is common in heart failure, the X-ROS mechanism is stronger and contributes to larger Ca^2+^, which might be arrhythmogenic [1,5]. The inclusion of length-dependent changes in troponin Ca^2+^ binding affinity lessens this response through increased buffering during stretch but will not abolish it completely. In other diseases such as Duchenne muscular dystrophy, X-ROS signaling has been suggested to play a role in triggering spontaneous Ca^2+^ release, which can lead to arrhythmias [1].

Another mechanism in which stretching can modify heart function is mechanoelectrical feedback. Mechanoelectrical feedback is thought to play a role in health and disease. In pacemaker cells, it has been suggested that the acute stretch cause by blood returning to the heart can lead to more rapid depolarization, increasing heart rate. In atria, stretching has been associated with sustained atrial fibrillation. In ventricles, stretch has been associated with increased arrhythmias [22]. X-ROS signaling might play a role here as well. Some ion channels have been shown to alter function due to ROS. Calcium has also been shown to feed back on the action potential. While both of these mechanisms are possible, further study is needed to determine if there is any significant role.

## 5. Conclusions

This study extends our previous work on the physiologic roles of X-ROS signaling in cardiac myocytes [5] and demonstrates how integrating experimental and modeling approaches can effectively help us dissect the mechanisms of Ca^2+^ dynamics in these cells. Our work yielded new insight into the interaction between X-ROS signaling and Ca^2+^ buffering by troponin; these insights could not have been realized with either approach alone. In the future, expanded models exploring the spatial aspects of X-ROS signaling are planned to better understand potential targets of X-ROS signaling and how X-ROS signaling may play a role in Ca^2+^ entrained arrhythmias.

## Data Availability

Model codes are publicly available at the Mason Archival Repository Service (MARS) at the following link: available online: https://hdl.handle.net/1920/11957 (accessed on 10 May 2021).
